# Mopane worm allergy in a 36-year-old woman: a case report

**DOI:** 10.1186/1752-1947-4-42

**Published:** 2010-02-06

**Authors:** Okechukwu A Okezie, Koloi K Kgomotso, Mavis M Letswiti

**Affiliations:** 1 Tshepo Clinic, Botswana Harvard Partnership, Hospital Road, Private Bag BO320, Gaborone, Botswana

## Abstract

**Introduction:**

The increasing incidence of new diseases as well as changing features of known diseases has partly been attributed to the impact of environmental changes. As a result, there have been calls from health experts for proper surveillance and monitoring of these changes.

This is a report of mopane worm allergy in a 36 year old female from the Tswana tribe in Botswana. Mopane worm, the caterpillar stage of Gonimbrasia belina moths, is a seasonal delicacy to people in many communities in southern Africa. As a result, by adulthood, many residents of these communities have had substantial exposure to the worm. Gonimbrasia belina moths belong to the Lepidoptera order of insects. Though some members of this order are known to induce contact allergy, there is no reported incidence of ingestion allergy from mopane worm. Therefore, it is important to track this case for its epidemiological significance and to alert both clinicians and the vulnerable public on the incidence of mopane worm allergy in this region.

**Case presentation:**

This is a case of a 36 year old woman from the Tswana ethnic group in Botswana, who was diagnosed with food allergy. She presented with itchy skin rash, facial swelling, and mild hypotension after eating mopane worm. She had no previous history of allergic reaction following contact or ingestion of mopane worm and had no atopic illness in the past. She was treated and her symptoms resolved after 4 days.

**Conclusion:**

The proper management of allergy involves patients' avoidance and clinicians' predictability. Though hypothetical, this report is expected to sensitize clinicians to anticipate and properly manage subsequent occurrence, as well as educate the public in these communities. In addition, tracking new disease patterns, with relationship to environmental changes, will compliment existing evidence in validating the importance of proper environmental surveillance and management.

## Introduction

In spite of the recent aversion in some developed countries, the eating of insects is still wide spread. Caterpillars and termites are the most eaten and marketed insects in Africa [1]. Among these are Mopane worms; caterpillars that hatch in early spring from eggs of Gonimbrasia belina moths. They are mostly seen on Colophospermum mopane trees where they mature within six weeks. Due to its high nutritional content, palatability, ease of processing and storage, mopane worm is an accessible nutritious supplement and a source of income for some people in Southern African communities [1].

In spite of widespread human contacts through seasonal harvesting and ingestion in these communities, there is no documented case of mopane worm allergy [2].

Food allergy, like other allergic conditions, is the result of the body's immune reactions to proteins, in this case, food proteins [3]. Immunological [IgE] association distinguishes food allergy from food intolerance which is the commonest food related reaction [4]

Food allergies like intolerance is more common in children due to early exposures to the trigger proteins before their immune system is matured enough to handle these proteins [4-6]. With maturity, most allergic responses to some proteins may wane or completely disappear [7].

The conscious selection of foods in the environment and avoidance of harmful substances is an evolutionary characteristic of living organisms, including man. There is usually a clear distinction between harmful and safe foods in a stable ecology.

As a result, apart from known idiosyncratic intolerance/allergies to known foods such as peanuts, tree nuts, milk, soy, wheat, eggs and sea foods, which account for about 90% of food allergies [7,8], unprecedented allergies from safe and uncontaminated foods are rare.

Elements of the environment are said to affect human health in two ways; through pathogenic agents and physical and chemical agents such as radiation, chemical compounds and emissions of gases, liquids or solids [9].

Over the last two decades, environmental activities and their impacts on life have raised concerns among health experts on future threats to human health [10-12].

The importance of maintaining the balance in the ecosystem was emphasized by Eric Chivian who elaborated the vital function of the ecosystem in regulating the concentration of oxygen, carbon dioxide and water vapour in the atmosphere, filtering pollutants from drinking water, regulating global temperature and precipitation, forming soil and keeping it fertile, pollinating plants, and providing food and fuel [13].

The rising incidence of microbial diseases such as plague, cholera, Lyme disease, and West Nile viral infections have been linked to microbial activities in response to global environmental changes [12].

Similarly, there is increasing global prevalence of allergic diseases due to ozone impact and new dietary habits, among other factors [10]

As a result, WHO and other public health experts have advised on proper public health surveillance and response to these emerging health threats, due to the impact of environmental changes [11-13].

Mopane worm ingestion is popular amongst most tribes in southern Africa. And recently, excessive harvesting in these regions has raised fears of the worm extinction [1].

The production of proteins and peptide, which are necessary for specie sustenance at the treat of extinction, is an adaptive characteristic of the insect species [10]. Though allergic reactions are also triggered by proteins, this report does not have the statistical strength to correlate the emergence of mopane worm allergy to adaptive response of these worms to selective pressure. However, by tracking this new case, which is significant to South African communities, a logical argument could be made for intensified surveillance and monitoring of health events from environmental manipulations, including presumed harmless activities in vulnerable communities.

## Case presentation

On 16/01/08, a 36 year old female presented to a Private Hospital in Botswana. She is a hospital nurse from the Tswana tribe in Botswana. She is married and has 2 children.

She complained of progressive body swelling that was worse on the face and generalized itchy body rash, malaise and nausea. She revealed ingestion of about 20 grams of mopane worm, 2 hours before the onset of symptoms. She had lived in Botswana all her life and had eaten mopane worm most of its season since childhood. She has had neither personal nor family history of similar symptoms or atopic syndrome. Except for occasional headache often attributed to tension headache, she had no significant medical history.

She had taken 4 mg of pediatric chlopheniramine syrup before going to the hospital.

According to the records from the accident and emergency of the hospital, her initial assessment revealed that she was anxious, but physically stable.

She had generalized maculopapular rash, normal systemic [cardio respiratory] function, mild hypotension.

Her vital signs were; Blood Pressure 90/50 mmHg; Pulse Rate 110/minutes; Respiratory Rate 14/minutes.

A diagnosis of food allergy was made.

She was treated with intramuscular injection of adrenalin followed by intravenous Hydrocortisone and promethazine through a venous line. The venous line was maintained until she received I liter of normal saline drip over 8 hours, while being observed.

She was discharged on oral chlopheniramine.

Two days later, she presented to our clinic with increasing rash, facial swelling, nausea, dizziness and yellowness of the eyes. There was no vomiting and patient reported a daily fluid intake of up to two and half liters and a good urine output.

She confirmed eating an unusually large quantity of mopane warm with the rest of the family members. She had been eating and tolerating the warm in most of the season since child hood.

On clinical examination, she had generalized macular exanthem, periorbital swelling, mild conjunctiva ingestion, pale extremities [palms] and dry lips.

Her vital signs were normal; Blood Pressure was 110/65 mmHg; Pulse Rate was 78/minutes; Respiratory Rate was 12/minutes. Examinations of the systems in the body were normal.

Her urine was tea colored but dipstick test was normal. Liver function test was also normal; White Blood Cell was marginally high at 11000 with predominant neutrophyls. Eosinophil ratio to other cells was normal.

Reaffirming the diagnosis of food allergy, we started her on high dose prednisolone and promethazine but discontinued chlopheniramine.

We advised her to drink lots of fluid, at least 3 liters daily, avoid the worm, and return if she didn't feel better. When called on 19/01/08, she said she felt much better.

On 21/01/08 she returned for assessment. She was well, so we stopped her promethazine and started tailing off her prednisolone. She was discharged and told to commence her routine activities on the 23/01/08.

## Conclusion

Relevant measures aimed at preserving the natural environment and forestalling emerging health consequences of environmental degradation have been prescribed. Nonetheless, the complexity of the earth's ecology and its activities, limits accurate prediction of these health impacts, and makes appropriate preventive solutions, elusive.

The effect of this limitation is more felt in developing country with competing priorities.

The rising incidence of allergic conditions among other diseases has been attributed to the disruption of ecosystem. Though it may be difficult at the clinic level to forestall this trend, accurate diagnosis and treatment is vital in tracking and reducing the impact of these diseases.

Food allergy is diagnosed from medical history, specific skin test and IgE identification [14].

With the clinical relevance of positive skin prick test and finding of food specific IgE limited by sensitization in patients, demonstration of a provoked allergy induced reaction in patient is the identified gold standard in diagnosis [14]

Management of allergic conditions like most medical condition in limited resource settings is guided by history, symptoms and signs. Care protocol is aimed at patient's comfort and satisfaction while conserving cost. Therefore, provocative tests are rarely done after symptom resolution has been achieved by empirical treatment. The target is usually to initiate treatment, monitor response, and confirm diagnosis on the basis of timing and association of symptoms to clinical presentation as well as response to treatment. Diagnosis is further supported by symptom relief if suspected cause is removed and reoccurrence of symptom if reintroduced [15]

Allergic reactions from pre exposed substance are usually identified earlier in individuals; hence, predictability makes it a relatively avoidable and manageable clinical condition in adults.

However, this case report challenges this premise and validates theories on immunologic dynamism from multiple factors such as disease, genetics environment, etc, which make accurate understanding of most related diseases and symptom spectrum elusive.

Also significant, is the speculation that the timing of the event in this report raises in the light of recent fears of mopane worm extinction from overhavesting. Anthropological reports support the theory that the imminent threat of extinction of a seasonal worm with a short life span can trigger rapid adaptive changes, which include production of protein with protective functions. These protective functions, among others, include unprecedented protein sensitization and food allergies that may range from reaction such as this case, to life threatening anaphylaxis and poisoning.

This speculation further supports the safe logic that planned activities towards reversing the harmful impact of disturbances in the ecosystem should be guided by proper understanding of natural activities in the system, in this case, the innate adaptive ability of organisms to selective pressures.

In emphasizing the importance of understanding the mutual biological and environmental modifications in global change for a sound policy decision and regulations, Dr. James M. Tiedje, Michigan State University, an author of the report who chairs ASM's Committee on Environmental Microbiology stated;

"We must better understand the human-microbe partnership so that environmental decisions that impact microbial processes will achieve appropriate balances in the atmosphere and biosphere. Otherwise, we will be increasingly challenged by unprecedented environmental problems," [11]

In the light of these, this report responds to the call for the tracking and monitoring of emerging disease patterns related to environmental changes.

It also gives interesting insights on the complexity of the interactions in the ecosystem, including specie adaptations and other activities. A thorough understanding, of which is vital for a comprehensive environmental management for health promotion.

Most importantly this original report raises a hypothesis that may motivate clinicians involvement in public health activities that includes environment surveillance and public education

## Abbreviations

ASM: American Society for Microbiology; mg: milligram; mmHg: millimeter mercury.

## Consent

Written informed consent was obtained from the patient for publication of this case report and accompanying images. A copy of the written consent is available for review by the Editor-in-Chief of this journal.

## Competing interests

The authors declare that they have no competing interests.

## Authors' contributions

ML performed the nursing care, retrieved and compiled this patient's personal and family medical records.

KK analyzed and interpreted all available medical data on food allergy from the hospital records and internet.

OO provided clinical care and was a major contributor in writing the manuscript.

All authors read and approved the final manuscript.
